# BET Bromodomain Inhibitors Suppress Inflammatory Activation of Gingival Fibroblasts and Epithelial Cells From Periodontitis Patients

**DOI:** 10.3389/fimmu.2019.00933

**Published:** 2019-04-30

**Authors:** Anna Maksylewicz, Agnieszka Bysiek, Katarzyna B. Lagosz, Justyna M. Macina, Malgorzata Kantorowicz, Grzegorz Bereta, Maja Sochalska, Katarzyna Gawron, Maria Chomyszyn-Gajewska, Jan Potempa, Aleksander M. Grabiec

**Affiliations:** ^1^Department of Microbiology, Faculty of Biochemistry, Biophysics and Biotechnology, Jagiellonian University, Kraków, Poland; ^2^Department of Periodontology and Oral Medicine, Faculty of Medicine, Jagiellonian University Medical College, Kraków, Poland; ^3^Department of Oral Immunology and Infectious Diseases, University of Louisville School of Dentistry, Louisville, KY, United States

**Keywords:** periodonditis, BET bromodomain, gingival fibroblast, gingival epithelial cell, porphyromonas gingivalis, I-BET151, chronic inflammation

## Abstract

BET bromodomain proteins are important epigenetic regulators of gene expression that bind acetylated histone tails and regulate the formation of acetylation-dependent chromatin complexes. BET inhibitors suppress inflammatory responses in multiple cell types and animal models, and protect against bone loss in experimental periodontitis in mice. Here, we analyzed the role of BET proteins in inflammatory activation of gingival fibroblasts (GFs) and gingival epithelial cells (GECs). We show that the BET inhibitors I-BET151 and JQ1 significantly reduced expression and/or production of distinct, but overlapping, profiles of cytokine-inducible mediators of inflammation and bone resorption in GFs from healthy donors (*IL6, IL8, IL1B, CCL2, CCL5, COX2*, and *MMP3*) and the GEC line TIGK (*IL6, IL8, IL1B, CXCL10, MMP9*) without affecting cell viability. Activation of mitogen-activated protein kinase and nuclear factor-κB pathways was unaffected by I-BET151, as was the histone acetylation status, and new protein synthesis was not required for the anti-inflammatory effects of BET inhibition. I-BET151 and JQ1 also suppressed expression of inflammatory cytokines, chemokines, and osteoclastogenic mediators in GFs and TIGKs infected with the key periodontal pathogen *Porphyromonas gingivalis*. Notably, *P. gingivalis* internalization and intracellular survival in GFs and TIGKs remained unaffected by BET inhibitors. Finally, inhibition of BET proteins significantly reduced *P. gingivalis*-induced inflammatory mediator expression in GECs and GFs from patients with periodontitis. Our results demonstrate that BET inhibitors may block the excessive inflammatory mediator production by resident cells of the gingival tissue and identify the BET family of epigenetic reader proteins as a potential therapeutic target in the treatment of periodontal disease.

## Introduction

Periodontitis is an inflammatory disease of the periodontium caused by microbial imbalance (dysbiosis) and the anaerobic bacterium *Porphyromonas gingivalis* plays a central role in driving the chronic inflammation (1). Oral pathogen interaction with gingival cells and infiltrating immune cells leads to a local immune response which fails to eradicate the invading bacteria which are equipped with sophisticated mechanisms of immune evasion. If unresolved, ongoing inflammation leads to periodontal ligament degradation, bone resorption and eventual tooth loss (2). Resident cells of the gingival tissue, including gingival epithelial cells (GECs) and gingival fibroblasts (GFs), represent the first line of defense against oral pathogens and are considered an important component of the innate immune system (3, 4). However, their chronic activation due to persistent interaction with oral bacteria, which involves the secretion of large quantities of cytokines, chemokines, matrix-degrading enzymes, and prostaglandins, significantly contributes to periodontitis pathogenesis (5).

Expression of inflammatory mediators is tightly regulated by epigenetic mechanisms, among which reversible acetylation of histone proteins plays a critical role. Importantly, pathological changes in histone acetylation and in expression of histone-modifying enzymes, histone acetyltransferases (HATs) and histone deacetylases (HDACs), have been identified in periodontitis patients and in a mouse model of periodontal disease (6, 7). Bromodomain proteins, 46 of which have been identified in the human genome, recognize ε-N-lysine acetylation motifs on histone tails and regulate the formation of acetylation-dependent chromatin complexes that are required for transcription (8). In particular, the ubiquitously expressed proteins BRD2, BRD3, BRD4, which belong to the bromodomain and extraterminal domain (BET) family, play distinct roles in coupling histone acetylation to gene transcription (9), including transcriptional activation of inflammatory genes (10). BET proteins are critical regulators of transcriptional elongation and cell division, and dysregulation of BET protein function, such as pathogenic chromosomal BRD4 translocations, has been identified in oncological conditions (11). BET proteins have thus emerged as potential therapeutic targets, and compounds targeting their tandem bromodomains are currently being evaluated in clinical trials (12).

The discovery of specific BET inhibitors acting as acetylated histone mimetics, I-BET151, and JQ1 (13, 14), has not only allowed for therapeutic targeting of BET proteins in cancer, but also provided insight into contributions of bromodomain-containing proteins to the pathogenesis of inflammatory disorders that are associated with an altered epigenetic landscape (15). BET inhibitors suppress lipopolysaccharide (LPS)- and cytokine-induced expression of inflammatory cytokines and chemokines in monocytes and macrophages *in vitro* and *in vivo*, and protect mice from lethal endotoxic shock and sepsis (16, 17). Inhibition of BET proteins also ameliorates inflammation and resulting pathology in animal models of several inflammatory diseases, including rheumatoid arthritis (RA), graft-vs. host disease and multiple sclerosis (18–20). Surprisingly, despite extensive efforts toward understanding bromodomain protein function in health and disease, little is still known about the role of BET proteins in the pathogenesis of periodontitis. The only study available to date demonstrated that the BET inhibitor JQ1 ameliorates gingival inflammation and alveolar bone destruction in *P. gingivalis*-induced experimental periodontitis in mice (21). In this model, the therapeutic effects of BET inhibition were attributed to diminished inflammatory cytokine production by macrophages and reduced osteoclast formation (21). However, the influence of JQ1 on other cell types involved in periodontitis pathogenesis has not been tested. In the present study, we investigated the effects of the BET bromodomain inhibitors I-BET151 and JQ1 on inflammatory and antimicrobial responses of resident cells of the gingival tissue, GFs and GECs, in the context of infection with the periodontal pathogen *P. gingivalis*.

## Materials and Methods

### Subjects, Cell Isolation, and Culture

Gingival tissue specimens for primary cell isolation were collected from healthy individuals undergoing orthodontic treatment (*n* = 9) and from patients with chronic periodontitis (*n* = 5) at the Department of Periodontology and Oral Medicine, Faculty of Medicine, Jagiellonian University Medical College in Kraków, Poland. This study was approved by and carried out in accordance with the recommendations of the Bioethical Committee of the Jagiellonian University in Kraków, Poland (permit numbers 122.6120.337.2016 and KBET/310/B/2012). All subjects gave written informed consent in accordance with the Declaration of Helsinki. Clinical characteristics of patients included in the study are shown in Supplementary Table 1.

The epithelial layer was separated enzymatically by treatment with dispase at 4°C overnight (o/n) and subjected to three rounds of digestion with trypsin (BioWest) for 10 min at 37°C. After centrifugation, the obtained GECs were suspended in keratinocyte growth medium (KGM-Gold, Lonza) and cultured in 6-well plates until confluence. GFs were isolated from the remaining connective tissue by digestion with 0.1% collagenase I (Invitrogen) at 37°C o/n. Cells were then vigorously pipetted, washed in PBS, suspended in Dulbecco's modified Eagle's medium (DMEM, Lonza) supplemented with 10% fetal bovine serum (FBS, EuroClone), 50 U/ml penicillin/streptomycin and 50 U/ml gentamicin, and cultured in T75 flasks. Cells were cultured in the presence of 10 μg/ml nystatin until passage 2 to prevent fungal contamination. The isolation procedure and the homogeneity of GF cultures have been standardized and described previously (22). GECs were used for experiments at passage 2 and GFs were used between passages 4 and 9. Telomerase-immortalized gingival keratinocytes (TIGKs, RRID:CVCL_M095) were kindly provided by Prof. Richard J Lamont (University of Louisville School of Dentistry) (23) and were cultured in KGM-Gold. One day prior to and during experiments, GFs were cultured in antibiotic-free DMEM containing 2% FBS, whereas TIGKs and primary GECs were cultured in antibiotic-free KGM-Gold.

### Bacterial Culture and Cell Infection

*Porphyromonas gingivalis* wild-type strain ATCC 33277 was grown on blood agar plates as described elsewhere (24). After anaerobic culture for 5–7 days at 37°C, bacteria were inoculated into brain–heart infusion (BHI) broth (Becton Dickinson) supplemented with 0.5 mg/ml L-cysteine, 10 μg/ml hemin and 0.5 μg/ml vitamin K, and cultured o/n in an anaerobic chamber (85% N_2_, 10% CO_2_, and 5% H_2_). Bacteria were then washed in PBS, resuspended in fresh BHI broth at optical density (OD)_600nm_ = 0.1 and cultured for ~20 h. A bacterial suspension at OD_600nm_ = 1 [corresponding to 10^9^ colony-forming units (CFU)/ml] in PBS was prepared and used for experiments. In some experiments, bacteria were heat-inactivated by 30 min incubation at 60°C or were treated with specific gingipain inhibitors KYT-1 and KYT-36 (Peptide Institute Inc.). 1μM KYT-1 and KYT-36 were incubated with bacteria for 20 min at 37°C prior to infection and were added to culture media for the duration of infection.

### RNA Isolation and Quantitative (q)PCR

GFs and TIGKs were seeded at 2.5 × 10^5^ cells per well in 12-well plates and after o/n culture were treated with DMSO [0.005% (V/V)] (BioShop), 1 μM I-BET151 (TargetMol) or 1 μM JQ1 (Abcam) for 30 min followed by stimulation with TNF, IL-1β (10 ng/ml, both from BioLegend) or infection with *P. gingivalis* at a multiplicity of infection (MOI) of 100 for 4 h. In some experiments, protein synthesis was blocked with 10 μg/ml cycloheximide (CHX, TargetMol). Total RNA was isolated using an EZ-10 Spin Column Total RNA Minipreps Super Kit (Bio-Basic), quantified using a Nanodrop spectrophotometer (Thermo Scientific) and equivalent amounts of RNA (500–1,000 ng) were converted to cDNA using a High-Capacity cDNA Reverse Transcription Kit (Applied Biosystems). qPCR reactions were performed on a CFX96 Touch™ Real-Time PCR Detection System (BIO-RAD) using PowerUp SybrGreen PCR mix (Applied Biosystems) and primers (Genomed S.A.) listed in Supplementary Table 2. The data were analyzed using the CFX Manager (BIO-RAD) and changes in mRNA expression were calculated relative to RPLP0 (ribosomal protein lateral stalk subunit P0) expression using the ΔΔCT method unless otherwise indicated.

### ELISA

GFs and TIGKs were seeded at 5.0 × 10^4^ cells per well in 48-well plates and after o/n culture were treated with DMSO [0.005% (V/V)], I-BET151 or JQ1 (both at 100 nM−1 μM) for 30 min before stimulation with TNF or IL-1β (10 ng/ml), or infection with heat-inactivated or KYT-treated *P. gingivalis* for 24 h. Alternatively, after treatment with inhibitors, GFs were infected with *P. gingivalis* at an MOI of 100 for 1 h. Cells were then washed 3 times with PBS and cultured in fresh medium containing DMSO, I-BET151, or JQ1 for another 24 h prior to collection of supernatants. Cell-free culture supernatants were collected and concentrations of IL-6, IL-8, CCL2, and CCL20 were determined using ELISA MAX kits (BioLegend) according to the manufacturer's instructions. Absorbance was measured using an Infinite M200 microplate reader (Tecan).

### Measurement of Cell Viability

Cell viability was assessed using the MTT (3-(4,5-dimethylthiazol-2-yl)-2,5-diphenyltetrazolium bromide, Sigma-Aldrich) reduction assay as described previously (25).

### Immunoblotting

Cell lysates were prepared in 1x Laemmli's buffer (2% SDS, 10% glycerol, 125 mM Tris-HCl, pH 6.8) and protein content was quantitated using the Bradford assay (BioShop). In some experiments, cells treated with the HDAC inhibitor suberoylanilide hydroxamic acid (SAHA, 5 μM) (Abcam) were used as a positive control. Equivalent amounts of protein were resolved by electrophoresis on 10 or 15% polyacrylamide gels and transferred to the Immobilon-P^SQ^ PVDF membranes (Millipore). Membranes were then blocked in 2% milk (BioShop) in TBS containing 0.1% Tween-20 (BioShop) (TBS/T), and incubated at 4°C o/n with primary antibodies recognizing acetylated lysine, acetyl-histone 3 (Ac-H3), H3, IκBα, phospho (p)-p38, p-ERK, p-p65 (all from Cell Signaling Technology), or tubulin (clone DM1A, Sigma-Aldrich). After washing in TBS/T, membranes were incubated with horseradish peroxidase (HRP)–conjugated anti-mouse or anti-rabbit Ig secondary antibodies (Dako) and developed with a Clarity Western ECL Substrate (BIO-RAD). Visualization was performed using a ChemiDocMP Imaging System and the ImageLab software (BIO-RAD).

### Measurement of Bacterial Internalization and Survival—Colony-Forming Assay

Cells were treated with 0.005% (V/V) DMSO or 1 μM I-BET151 in triplicate wells for 20 h prior to infection with *P. gingivalis* at an MOI of 100 for 1 h. Cells were then washed 3 times with PBS and were either lysed immediately in sterile distilled water for 20 min or cultured in fresh medium containing DMSO or I-BET151 for another 24 h before lysis. Cell lysates were serially diluted and 10μl of each dilution was plated in duplicate on blood agar plates and cultured for 5–7 d anaerobically at 37°C. Bacterial colonies were counted and the data were expressed as CFU per cell.

### Statistical Analyses

Data are presented as the mean +SEM. In studies of primary GFs and GECs, n's represent cell lines from individual donors/patients, while in TIGK studies n's refer to independent experiments. Parametric tests were used for comparisons between groups (ratio paired *t*-test or one-way ANOVA followed by Bonferroni multiple comparison test, where appropriate). *p* < 0.05 were considered statistically significant.

## Results

### BET Inhibitors Suppress Cytokine-Induced Inflammatory Activation of GFs and TIGKs

**Figure 1:**
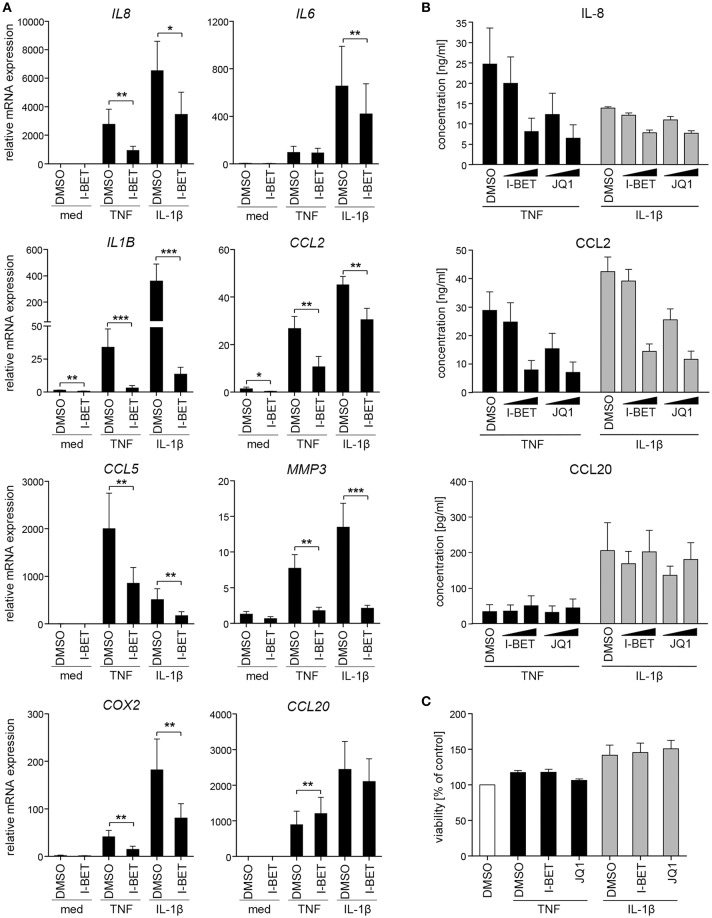
BET inhibitors suppress cytokine-induced gingival fibroblast (GF) inflammatory activation without affecting cell viability. **(A)** Relative mRNA expression of *IL8, IL6, IL1B, CCL2, CCL5, MMP3, COX2*, and *CCL20* in GFs treated with DMSO or 1 μM I-BET151 for 30 min prior to simulation with TNF or IL-1β (both at 10 ng/ml) for 4 h analyzed by qPCR (mean + SEM; *n* = 4–5). *P < 0.05, **P < 0.01, ***P < 0.001; ratio paired *t*-test. **(B)** IL-8, CCL2, and CCL20 production by GFs treated with DMSO or I-BET151 or JQ1 at two different concentrations (100 nM and 1 μM) for 30 min prior to simulation with TNF or IL-1β (both 10 ng/ml) for 24 h determined by ELISA (mean concentration + SEM; *n* = 5). **(C)** Viability of GFs (*n* = 3) treated with DMSO, I-BET151, or JQ1 (both at 1 μM) for 30 min prior to simulation with 10 ng/ml TNF or IL-1β for 24 h assessed using MTT assay and presented as % of control + SEM.

We initiated this study by investigating whether BET bromodomain proteins are involved in GF inflammatory activation in response to cytokines that are present in the inflamed gingival tissue. Primary GFs from healthy individuals were stimulated with TNF or IL-1β in the presence of the BET inhibitor I-BET151. BET inhibition significantly suppressed cytokine-induced mRNA expression of a broad range of inflammatory mediators involved in the pathogenesis of periodontitis, including *IL8, IL1B, CCL2, CCL5, MMP3*, and *COX2* (Figure 1A). Interestingly, some selectivity of I-BET151 effects on GF gene expression was noted, as I-BET151 suppressed IL-1β-, but not TNF-induced *IL6* expression, and, surprisingly, promoted expression of the antimicrobial chemokine *CCL20* in response to TNF stimulation. In the absence of inflammatory stimulation, I-BET151 significantly reduced mRNA levels of *IL1B* and *CCL2*, whereas expression of other mediators included in our analyses remained unaffected (Figure 1A). Next, to test if effects of BET inhibition on mRNA expression translate into changes in protein levels, we treated GFs with I-BET151 or a chemically unrelated BET inhibitor, JQ1, prior to 24 h cytokine stimulation. Both compounds dose-dependently suppressed TNF- and IL-1β-induced IL-8 and CCL2 production, reaching up to 70% inhibition at 1 μM. JQ1 displayed more potent inhibitory activity, significantly suppressing IL-8 and CCL2 secretion already at 100 nM (Figure 1B). In contrast, BET inhibition had no significant effects on agonist-induced CCL20 production, though a trend toward increased CCL20 secretion in the presence of TNF stimulation was noted (Figure 1B), consistent with mRNA data. To exclude the possibility that the observed effects of BET inhibitors on GF activation could be attributed to compound cytotoxicity, GFs were exposed to I-BET151 or JQ1 in the presence of cytokine stimulation for 24 h. Both compounds had negligible effects on cell viability as determined using the MTT reduction assay (Figure 1C).

**Figure 2:**
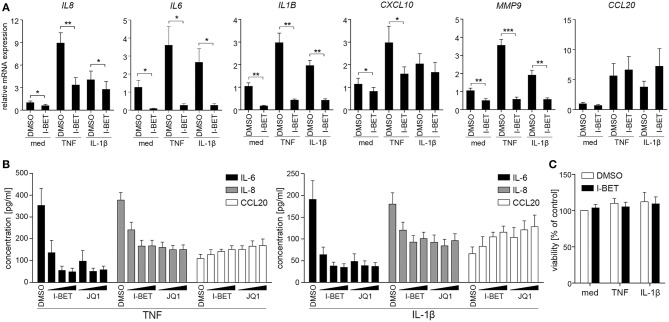
BET inhibitors reduce cytokine-induced inflammatory mediator production by telomerase-immortalized gingival keratinocytes (TIGKs) without affecting cell viability. **(A)** Relative mRNA expression of *IL8, IL6, IL1B, CXCL10, MMP9*, and *CCL20* in TIGKs treated with DMSO or 1 μM I-BET151 for 30 min prior to simulation with TNF or IL-1β (both at 10 ng/ml) for 4 h determined by qPCR (mean + SEM; *n* = 4–5). *P < 0.05, **P < 0.01, ***P < 0.001; ratio paired *t*-test. **(B)** IL-6, IL-8, and CCL20 production by TIGKs treated with DMSO or increasing concentrations (100 nM, 500 nM, and 1 μM) of I-BET151 or JQ1 for 30 min followed by simulation with 10 ng/ml TNF (left panel) or 10 ng/ml IL-1β (right panel) for 24 h measured by ELISA (mean concentration + SEM; *n* = 5). **(C)** Viability of TIGKs (*n* = 3) treated with DMSO or 1 μM I-BET151 for 30 min prior to simulation with TNF or IL-1β (both 10 ng/ml) for 24 h analyzed using MTT assay and shown as % of control + SEM.

To verify whether BET inhibitors similarly modulate inflammatory responses of GECs, we utilized TIGKs, an immortalized cell line derived from healthy donor GECs that closely mimics primary cell responses (23). TNF and IL-1β stimulation upregulated a cluster of inflammatory mediators in TIGKs that was distinct from, but partly overlapping with that observed in GFs. I-BET151 treatment significantly suppressed cytokine-induced mRNA expression of *IL8, IL6, IL1B, CXCL10*, and *MMP9* (Figure 2A). Interestingly, BET inhibition reduced not only inducible, but also basal levels of these transcripts. Similar to observations in GFs, BET inhibition had limited effect on *CCL20* expression. Interestingly, the sensitivity of individual genes to transcriptional suppression by I-BET151 differed between the two analyzed cell types: while I-BET151 reduced cytokine-induced *IL6* mRNA accumulation by >90% in TIGKs (Figure 2A), it had modest effects on *IL6* expression in GFs (Figure 1A), indicating differential requirements for BET proteins in transcriptional induction of the same gene in two different cell types. The observed effects of BET inhibition were next confirmed at the protein level. I-BET151 and JQ1 dose-dependently reduced IL-6 and IL-8 accumulation in cell culture supernatants, whereas CCL20 production was moderately induced (Figure 2B). Maximum suppression of IL-6 and IL-8 production by BET inhibitors was achieved at 100-500 nM, suggesting that TIGKs might be more sensitive to low concentrations of BET inhibitors compared to GFs. In line with previous observations in GFs, I-BET151, and JQ1 had no effect on TIGK viability (Figure 2C).

### BET Inhibitors Have No Effect on Inflammatory Signaling Pathway Activation in GFs and Do Not Require New Protein Synthesis for Gene Suppression

**Figure 3:**
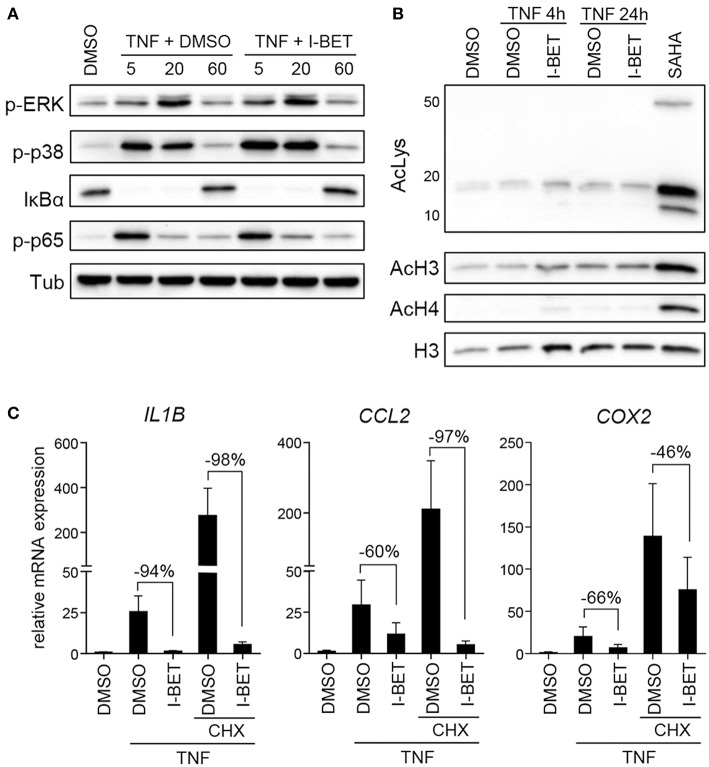
I-BET151 has no effect on MAPK and NFκB activation in gingival fibroblasts (GFs) and new protein synthesis is not required for inflammatory gene suppression by I-BET151. **(A)** Western blot analysis of phospho (p)-ERK, p-p38, IκBα, p-p65(Ser536), and tubulin (Tub) in total cell lysates of GFs after 30 min treatment with DMSO or 1 μM I-BET151 followed by stimulation with 10 ng/ml TNF for 5, 20, or 60 min. **(B)** Western blot analysis of total acetylated lysine (AcLys), acetyl-histone H3(Lys18) (AcH3), acetyl-H4(Lys8) (AcH4), and total H3 in cell lysates of GFs treated with DMSO or 1 μM I-BET151 for 30 min prior to TNF (10 ng/ml) stimulation for 4 or 24 h. Cells treated with SAHA for 4 h were used as a positive control for lysine hyperacetylation. Data representative of 2–3 independent experiments are shown in **(A,B)**. **(C)** Relative mRNA expression of *IL1B, CCL2*, and *COX2* in GFs treated with DMSO or 1 μM I-BET151 in the presence or absence of cycloheximide (CHX) for 30 min prior to simulation with 10 ng/ml TNF for 4 h analyzed by qPCR (mean + SEM; *n* = 4; % of suppression compared to DMSO control are depicted in each graph).

Inhibition or silencing of BET proteins regulates gene expression not only through disruption of interactions between bromodomains and acetylated histones at individual gene promoters, but also by affecting acetylation-dependent signaling pathways, including mitogen-activated protein kinase (MAPK) and NF-κB signaling (26, 27). We therefore analyzed the effects of BET inhibition on TNF-induced activation of signaling pathways critical for inflammatory cell activation and on protein acetylation status. Treatment of primary GFs from healthy donors with I-BET151 had no effect on p38 and extracellular signal-regulated kinase (ERK) MAPK activation, degradation of IκBα and phosphorylation of p65 NFκB subunit (Figure 3A). I-BET151 also failed to affect histone H3 and H4 acetylation, as well as the levels of total acetylated lysine detected in cellular lysates (Figure 3B), excluding the possibility that BET inhibitors could affect GF activation through off-target effects on HATs or HDACs. Finally, we tested whether BET inhibitors could affect gene expression indirectly by induction of gene repressors in GFs. I-BET151 suppressed *IL1B, CCL2*, and *COX2* expression both in the absence or presence of the protein synthesis inhibitor CHX (Figure 3C). Suppression of *IL6* and *IL8* by I-BET151 in TNF-stimulated TIGKs was also unaffected by CHX (Supplementary Figure 1), indicating that *de novo* synthesis of repressor proteins is not necessary for suppression of inflammatory mediators by BET inhibitors in both cell types.

### BET Inhibitors Suppress Inflammatory Mediator Production by GFs and TIGKs Infected With *P. gingivalis*

**Figure 4:**
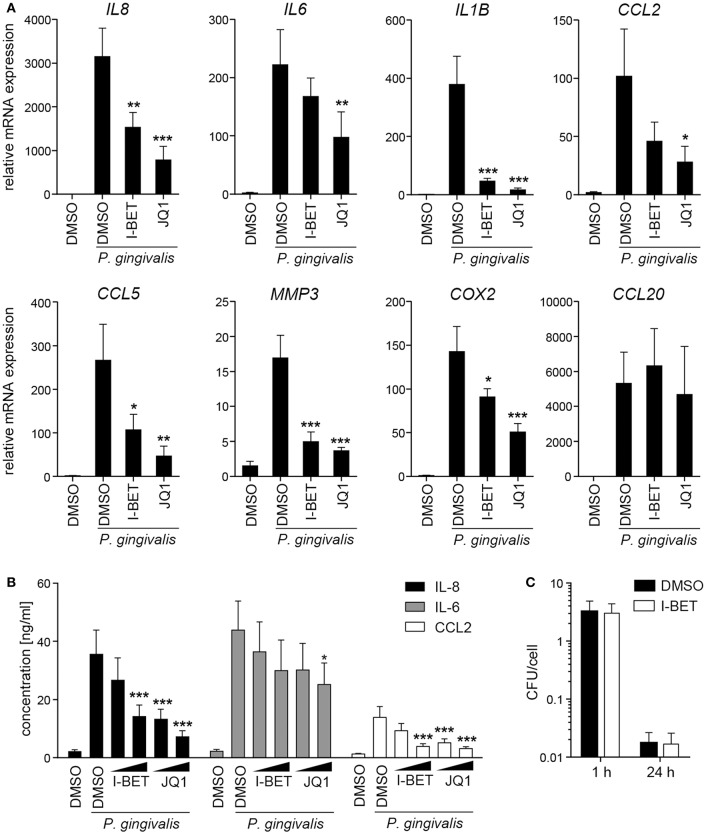
BET inhibitors suppress production of inflammatory mediators by gingival fibroblasts (GFs) infected with *P. gingivalis*. **(A)** qPCR analysis of relative mRNA expression of *IL8, IL6, IL1B, CCL2, CCL5, MMP3, COX2*, and *CCL20* in GFs treated with DMSO, I-BET151 or JQ1 (both at 1 μM) for 30 min prior to infection with *P. gingivalis* (MOI = 100) for 4 h (mean + SEM; *n* = 5–6). **(B)** Production of IL-8, IL-6 and CCL2 by GFs exposed to DMSO or I-BET151 or JQ1 at two different concentrations (100 nM and 1 μM) for 30 min before *P. gingivalis* infection (MOI = 100) for 1 h, followed by washing and 24 h culture in fresh medium containing DMSO or BET inhibitors (mean concentration + SEM; *n* = 5–6). **(A,B)** *P < 0.05, **P < 0.01, ***P < 0.001; one-way ANOVA followed by Bonferroni multiple comparison test. **(C)** Intracellular survival of *P. gingivalis* in GFs treated with DMSO or 1 μM I-BET151 for 20 h prior to infection with *P. gingivalis* (MOI = 100) for 1 h determined by colony-forming assay immediately (1 h) or 24 h post-infection. Data are presented as mean colony-forming units (CFU)/cell + SEM of 4 independent experiments.

In periodontal disease, *P. gingivalis* directly interacts with and invades resident cells of the gingival tissue, which not only contributes to the chronicity of inflammation, but also facilitates bacterial spreading to deeper tissues. To analyze the role of BET proteins in GF and TIGK activation and antimicrobial responses, cells were treated with I-BET151 or JQ1 prior to infection with *P. gingivalis*. In primary GFs from healthy donors, induction of inflammatory mediator expression by *P. gingivalis* was comparable to, or even higher than that observed following cytokine stimulation, and BET inhibition significantly suppressed bacteria-induced upregulation of *IL8, IL1B, CCL2, CCL5, MMP3*, and *COX2*, but not *CCL20* (Figure 4A). In line with previous observations, JQ1 was more potent in blocking gene induction than I-BET151. Although the kinetics of mRNA induction by *P. gingivalis* differed between individual genes, I-BET151 uniformly reduced *IL8, IL1B, CCL2, and CCL5* expression at 4 and 24 h post-infection (Supplementary Figure 2). Production of IL-8 and CCL2 protein by *P. gingivalis*-infected GFs was also dose-dependently reduced by both BET inhibitors, whereas suppression of IL-6 production was less pronounced (Figure 4B). Next, to test whether BET inhibitors affect cellular response to *P. gingivalis* infection, GFs were treated with I-BET151 for 20h prior to infection with *P. gingivalis* for 1 h, and the presence of live bacteria in cell lysates was determined using the colony-forming assay. I-BET151 had no effect on the numbers of bacteria detected in GFs immediately and 24 h post-infection (Figure 4C), indicating that BET proteins are not involved in internalization and elimination of intracellular *P. gingivalis* by GFs.

**Figure 5:**
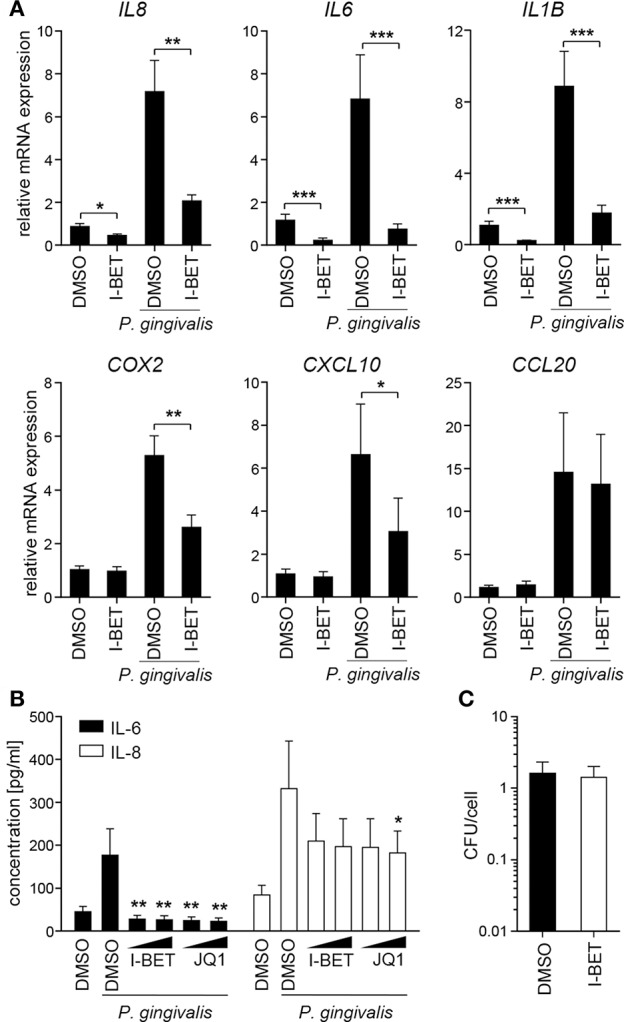
BET inhibitors block inflammatory activation of telomerase-immortalized gingival keratinocytes (TIGKs) infected with *P. gingivalis*. **(A)** Relative mRNA expression of *IL8, IL6, IL1B, COX2, CXCL10*, and *CCL20* in TIGKs cultured for 30 min with DMSO or I-BET151 (1 μM) followed by 4 h infection with *P. gingivalis* (MOI = 100) determined by qPCR (mean + SEM; *n* = 4–5). *P < 0.05, **P < 0.01, ***P < 0.001; ratio paired *t*-test. **(B)** Production of IL-6 and IL-8 by TIGKs treated with DMSO or I-BET151 or JQ1 at two different concentrations (100 nM and 1 μM) for 30 min prior to infection with heat-inactivated *P. gingivalis* (MOI = 100) for 24 h (mean concentration + SEM; *n* = 4). *P < 0.05, **P < 0.01; one-way ANOVA followed by followed by Bonferroni multiple comparison test. **(C)** Intracellular survival of *P. gingivalis* in TIGKs treated with DMSO or 1 μM I-BET151 for 20 h before infection with *P. gingivalis* (MOI = 100) for 1 h determined by colony-forming assay immediately after infection. Results are presented as mean colony-forming units (CFU)/cell + SEM of 4 independent experiments.

Infection of TIGKs with *P. gingivalis* also led to transcriptional upregulation of a set of inflammatory genes largely overlapping with that induced by cytokines (Figure 5A). I-BET151 treatment significantly reduced mRNA levels of *IL8, IL6, IL1B, COX2*, and *CXCL10*, while leaving *CCL20* expression unaffected (Figure 5A). I-BET151 and JQ1 also significantly reduced TIGK IL-6 and IL-8 production induced by exposure to heat-inactivated *P. gingivalis*. Both compounds inhibited IL-6 and IL-8 secretion by ~85 and 40%, respectively, already at 100 nM (Figure 5B). A similar degree of IL-6 and IL-8 inhibition by BET inhibitors was observed in TIGKs infected with live *P. gingivalis* in the presence of gingipain inhibitors that were used to eliminate the potential confounding factor of cytokine degradation by bacterial proteases (data not shown). Importantly, BET inhibitor effects were restricted to inflammatory gene transcription as treatment with I-BET151 for 20h prior to infection failed to affect *P. gingivalis* internalization by TIGKs (Figure 5C), consistent with previous observations in GFs. Collectively, these results demonstrate that BET inhibitors suppress production of a broad range inflammatory mediators by GFs and GECs without affecting *P. gingivalis* internalization and survival within the infected cells.

### BET Inhibitors Reduce Inflammatory Gene Expression in GFs and GECs From Patients With Periodontitis

**Figure 6:**
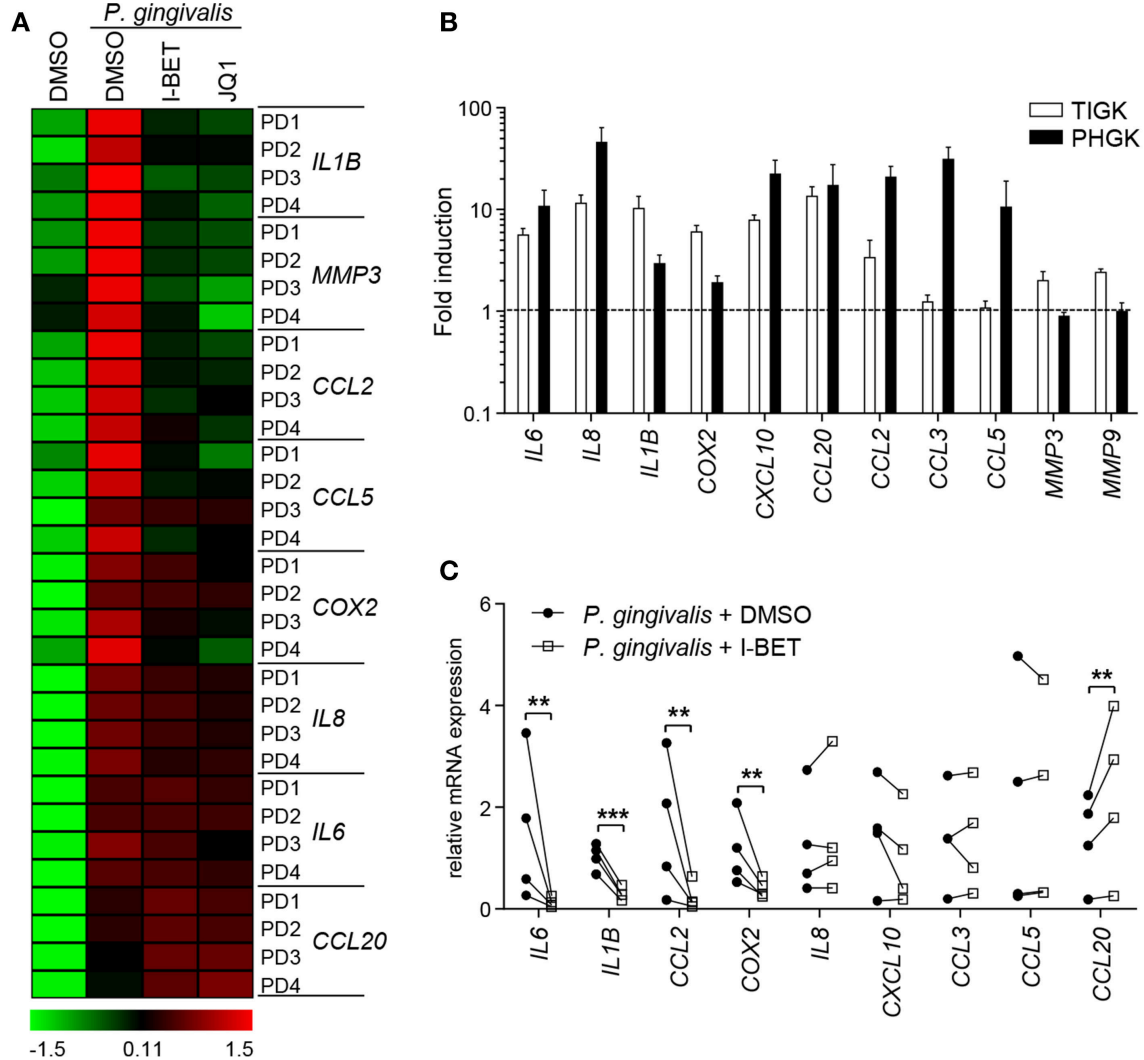
Gingival fibroblasts (GFs) and gingival epithelial cells (GECs) from periodontitis patients are sensitive to the anti-inflammatory activity of BET inhibitors. **(A)** Primary GFs from patients with periodontitis were treated with DMSO, I-BET151 or JQ1 (both at 1 μM) for 30 min prior to infection with *P. gingivalis* (MOI = 100) for 4 h and mRNA levels of inflammatory mediators were determined by qPCR. Data for individual patients (PD1–PD4) are presented on a heat map as row *Z*-scores calculated from ΔCt values relative to a housekeeping gene (RPLP0). **(B)** Comparison of inflammatory mediator induction in TIGKs and primary GECs from periodontitis patients after 4 h infection with *P. gingivalis* (MOI = 100) (mean fold induction compared to uninfected cells + SEM; *n* = 3 for TIGKs vs. *n* = 3 GEC lines from individual patients (PD1, PD2, PD5). **(C)** qPCR analysis of mRNA levels of inflammatory mediators in primary GECs from patients with periodontitis (PD1, PD2, PD3, PD5) treated with DMSO or 1 μM I-BET151 for 30 min followed by infection with *P. gingivalis* (MOI = 100) for 4 h. Symbols represent relative expression values from individual patients (*n* = 4). **P < 0.01, ***P < 0.001; ratio paired *t*-test.

GFs present in the inflamed gingival tissue in periodontitis patients display an activated phenotype and hyperresponsiveness to *P. gingivalis* infection *in vitro* (28, 29). We therefore examined whether GFs isolated from patients with periodontitis were resistant to the anti-inflammatory effects of BET inhibitors. The degree of suppression of *IL8, IL1B, CCL2, CCL5, MMP3*, and *COX2* caused by I-BET151 or JQ1 in *P. gingivalis*-infected GFs from periodontitis patients was comparable to that observed in healthy donor GFs (Figure 6A). JQ1, but not I-BET151, moderately reduced *IL6* mRNA levels, consistent with observations in GFs from healthy individuals, while *CCL20* expression was upregulated by both BET inhibitors (Figure 6A). Next, to assess the sensitivity of GECs from periodontitis patients to BET inhibitors, we first compared the inflammatory gene expression profile induced by *P. gingivalis* in TIGKs and primary GECs. While a shared cluster of genes that included *IL6, IL8, IL1B, COX2, CXCL10, CCL20*, and *CCL2* was upregulated by *P. gingivalis* in both types of cells, we also noted some differential responses. The chemokines *CCL3* and *CCL5* were selectively induced in GECs, but not in TIGKs, whereas expression of the metalloproteinases *MMP3* and *MMP9* was upregulated upon *P. gingivalis* infection only in TIGKs (Figure 6B). Treatment of primary GECs from periodontitis patients with I-BET151 prior to *P. gingivalis* infection resulted in significant suppression of *IL6, IL1B, CCL2*, and *COX2*. However, in contrast to TIGKs, *IL8*, and *CXCL10* mRNA levels were not affected by BET inhibition, whereas *CCL20* expression was consistently induced (Figure 6C). Together, these observations suggest that BET proteins play a key role in regulating inflammatory mediator expression in GFs and GECs in response to an oral pathogen.

## Discussion

A severe form of periodontitis affects approximately 10% of the human population, leading to inevitable tooth loss if left untreated, and is strongly associated with increased risk of developing systemic diseases, including RA, atherosclerosis and cancer (1, 30). Non-surgical treatment strategies conventionally used to treat periodontitis, scaling and root planing, or root surface debridement, focus solely on reducing bacterial challenge, but in many patients are insufficient to fully resolve chronic inflammation. Because of that, there is a growing need for identifying novel therapeutic strategies ameliorating the host inflammatory response that could be used as an adjunct to conventional treatment (31). The identification of anti-inflammatory properties of HDAC and BET inhibitors has generated great interest in the therapeutic potential of targeting epigenetic mechanisms in many inflammatory and infectious disorders, including periodontitis (32–34). Indeed, pharmacological modulators of histone acetylation protected against pathology in animal models of periodontal disease (21, 35). In particular, the BET inhibitor JQ1 has shown potent anti-inflammatory and bone-protective activity (21). Here, we show for the first time that BET bromodomain proteins are important regulators of resident gingival cell activation in response to *P. gingivalis* and inflammatory cytokines, and that small molecule BET inhibitors suppress production of cytokines, chemokines, and other mediators of inflammation by GFs and GECs from periodontitis patients.

Destruction of the gingival connective tissue, periodontal ligament, and alveolar bone in periodontitis is a consequence of a futile attempt by the host immune response to eradicate microbial pathogens (2). Resident gingival cells contribute to this vicious circle of chronic inflammation by secreting a plethora of inflammatory mediators, including cytokines (IL-6, IL-1β), chemokines (IL-8, CCL2, CCL5, CXCL10, CCL20), matrix-degrading enzymes (MMP1, MMP3, MMP9), and prostaglandins (5). Elevated levels of these mediators are present in gingival tissue and/or gingival crevicular fluid from periodontitis patients, correlating with disease severity (5, 36, 37), and studies in animal models show that genetic ablation or pharmacological targeting of IL-6 or IL-1β ameliorates pathology in experimental periodontitis (38). Our data provide evidence that BET proteins are required for transcriptional induction of key inflammatory molecules in GFs and GECs infected with *P. gingivalis* and thus represent a potential therapeutic target for modulation of the host immune response. Although quantitative differences in the sensitivity of individual genes to BET inhibition were noted, our observations are consistent with the broad anti-inflammatory activity of BET inhibitors reported in synovial fibroblasts and in airway epithelial cells (39, 40). While our data suggest that suppression of GF and GEC inflammatory activation by BET inhibitors is stimulus-independent, future studies should analyze the role of BET proteins in gingival cell response to a complex dental biofilm containing other oral pathogens, such as *Fusobacterium nucleatum, Tanerella forsythia*, and *Treponema denticola*. In this regard it is noteworthy that JQ1 attenuated gastric inflammation and immune cell infiltration in mice infected with *Helicobacter pylori* (41), indicating its immunosuppressive potential in chronic infections caused by Gram-negative pathogenic bacteria.

BET proteins regulate cellular activation not only at the gene promoter level, but also through chromatin-independent mechanisms. BRD4 binds acetylated lysine-310 of the p65 NF-κB subunit, enhancing its transcriptional activity and thus acting as a coactivator of NF-κB-dependent genes (26). In macrophages, BRD4 also modulates NF-κB signaling through translational control of IκBα resynthesis (42), whereas in endothelial cells BET inhibition blocks early events in NF-κB activation as well as p38 and JNK MAPK phosphorylation (27). These mechanisms are, however, cell type-specific and apparently not operational in GFs based on our analyses of MAPK, IκBα, and NF-κB p65 activation. While it remains to be determined if BET inhibition also fails to affect NF-κB transcriptional activity and binding to specific gene promoters in GFs, our findings are consistent with studies of synovial fibroblasts from RA patients demonstrating the lack of effect of I-BET151 on NF-κB and MAPK signaling pathways (40). We also show that *de novo* synthesis of repressor proteins is not required for suppression of inflammatory genes by I-BET151 in GFs and GECs, in line with observations in human macrophages (17). Collectively, these results argue against indirect or signaling pathway-dependent effects of BET inhibition in gingival cells and suggest that interference with direct interaction of BET proteins with acetylated histones at gene promoters is most likely responsible for the observed suppression of inflammatory mediator transcriptional induction. Although potential off-target effects of BET inhibitors cannot be ruled out based on our studies, extensive screening of these compounds failed to identify any significant effects on non-BET protein targets (13, 43).

Among the analyzed inflammatory genes, expression of *CCL20* was selectively upregulated by I-BET151 and JQ1 in some experimental conditions, particularly in GECs. CCL20 may play a dual role in periodontitis. As a chemoattractant for immature dendritic cells and Th17 cells, CCL20 may contribute to the chronicity of inflammation by facilitating immune cell recruitment and accumulation (37). On the other hand, CCL20 displays direct microbicidal activity against Gram-positive and Gram-negative bacteria through its antimicrobial regions structurally related to human β-defensin-2 (44). *CCL20* induction by BET inhibitors could therefore facilitate bacterial elimination by innate immune mechanisms. It remains to be verified if CCL20 exerts antimicrobial activity against *P. gingivalis* or other oral pathogens, and whether this mechanism is operational in the inflamed gingival tissue during periodontitis. While the exact role of bromodomain proteins in CCL20 regulation has yet to be investigated, HDAC inhibitors have also been shown to promote CCL20 production by GECs and intestinal epithelial cells (45, 46), suggesting a critical role for histone acetylation in CCL20 transcriptional regulation that requires detailed studies at the gene promoter level.

The idea that BET inhibitors may display clinical activity in periodontal disease is also supported by recent evidence that N-methyl-2-pyrrolidone (NMP), a component of dental barrier membranes used in dental procedures, displays bromodomain inhibitory activity (47). While initially thought to lack biological activity, it was later discovered that NMP displays anti-inflammatory and anti-osteoclastogenic activity similar to the effects of BET inhibitors (48, 49). The efficacy of NMP-based dental barrier membranes in periodontal tissue regeneration could therefore be partly attributed to their ability to inhibit BET bromodomains (50), a possibility that should be addressed in future studies directly comparing NMP and BET inhibitor activity in periodontitis models. These observations, together with the results of our study and the initial proof of principle obtained in an animal model (21), indicate that specific targeting of epigenetic reader proteins from the BET family may block the excessive inflammatory mediator production by multiple cell types important in the pathogenesis of periodontitis and reduce the significant morbidity associated with periodontal disease.

## Ethics Statement

This study was approved by and carried out in accordance with the recommendations of the Bioethical Committee of the Jagiellonian University in Kraków, Poland (permit numbers 122.6120.337.2016 and KBET/310/B/2012). All subjects gave written informed consent in accordance with the Declaration of Helsinki.

## Author Contributions

AM, AB, KBL, JMM, and GB contributed to research design, performed experiments, and analyzed data. MK obtained all clinical materials and analyzed clinical records. MS, KG, MC-G, and JP contributed to research design, data interpretation, and writing the manuscript. AMG designed the study, analyzed and interpreted the data, and wrote the manuscript. All authors contributed to manuscript revision, read and approved the submitted version.

### Conflict of Interest Statement

The authors declare that the research was conducted in the absence of any commercial or financial relationships that could be construed as a potential conflict of interest.
