# Supplementary material for: BET Bromodomain Inhibitors Suppress Inflammatory Activation of Gingival Fibroblasts and Epithelial Cells From Periodontitis Patients
Source: Front Immunol. 2019 Apr 30;10:933. doi: 10.3389/fimmu.2019.00933 (PMC6503739; doi:10.3389/fimmu.2019.00933)
Supplement: Supplementary file 1 [file Data_Sheet_1.PDF]

Supplementary Material

BET bromodomain inhibitors suppress inflammatory activation of gingival fibroblasts and epithelial cells from periodontitis patients.

Anna Maksylewicz, Agnieszka Bysiek, Katarzyna B Lagosz, Justyna M Macina, Malgorzata Kantorowicz, Grzegorz Bereta, Maja Sochalska, Katarzyna Gawron, Maria Chomyszyn-Gajewska, Jan Potempa and Aleksander M Grabiec

Correspondence:

Aleksander M Grabiec: aleksander.grabiec@uj.edu.pl, Jan Potempa: jan.potempa@uj.edu.pl

1. Supplementary Tables

Supplementary Table 1. Clinical characteristics of patients with periodontitis (n = 5) included in the study:

Characteristic	Median (range)
Age (y)	60 (49-60)
Male:female (n)	1:4
API [%]	33 (27.3-97)
SBI [%]	34.3 (24-74)
PPD [mm]	3.5 (1.3-4.5)
CAL [mm]	5.6 (4.6-7)

*API: approximal plaque index; SBI: sulcus bleeding index; PPD: probing pocket depth; CAL: clinical attachment loss.

Supplementary Table 2. Sequences of primers used for qPCR analyses.

Gene	Forward primer	Reverse primer
IL6	GACAGCCACTCACCTCTTCA	CCTCTTTGCTGCTTTCACAC
IL8	GCTCTGTGTGAAGGTGCAGT	CCAGACAGAGCTCTCTTCCA
IL1B	ACAGATGAAGTGCTCCTTCCA	GTCGGAGATTCGTAGCTGGAT
CCL2	TCTGTGCCTGCTGCTCATAG	GGGCATTGATTGCATCTGGC
CCL3	TGCTCAGAATCATGCAGGTCT	GCAGCAAGTGATGCAGAGAAC
CCL5	ATCCTCATTGCTACTGCCCTC	GCCACTGGTGTAGAAATACTCC
CCL20	AAGAGTTTGCTCCTGGCTGCTT	GCAGTCAAAGTTGCTTGCTGCT
COX2	AGCCCTTCCTCCTGTGCCT	AATCAGGAAGCTGCTTTTTACCT
CXCL10	TGAAATTATTCCTGCAAGCCAA	CAGACATCTCTTCTCACCCTTCTTT
MMP3	GAGGACACCAGCATGAACCT	CACCTCCAGAGTGTCGGAGT
MMP9	CCTGGAGACCTGAGAACCAAT	GCCACCCGAGTGTAACCATAG
RPLP0	GCGTCCTCGTGGAAGTGACATCG	TCAGGGATTGCCACGCAGGG

2. Supplementary Figures.

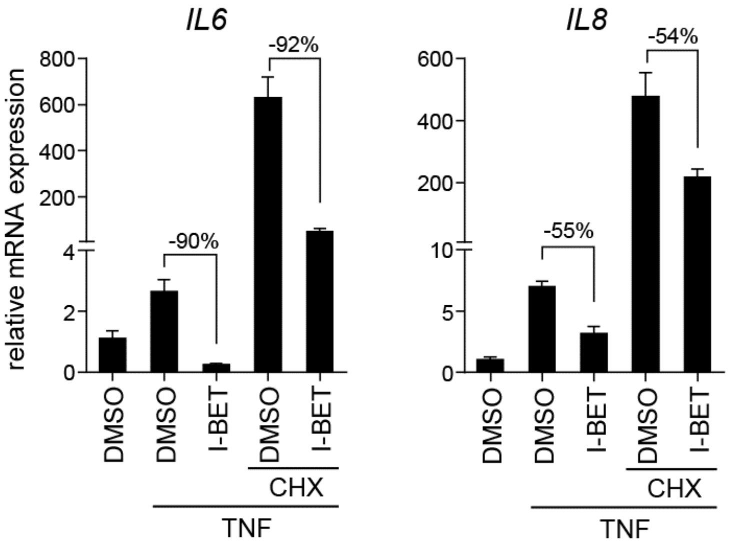

Supplementary Figure 1. New protein synthesis is not required for inflammatory gene suppression by I-BET151 in TIGKs. Relative mRNA expression of *IL6* and *IL8* in TIGKs treated with DMSO or 1 µM I-BET151 in the presence or absence of cycloheximide (CHX, 10 µg/ml) for 30 min prior to simulation with 10 ng/ml TNF for 4 h analyzed by qPCR (mean +SEM; n=4; % of suppression compared to DMSO control are depicted in each graph).

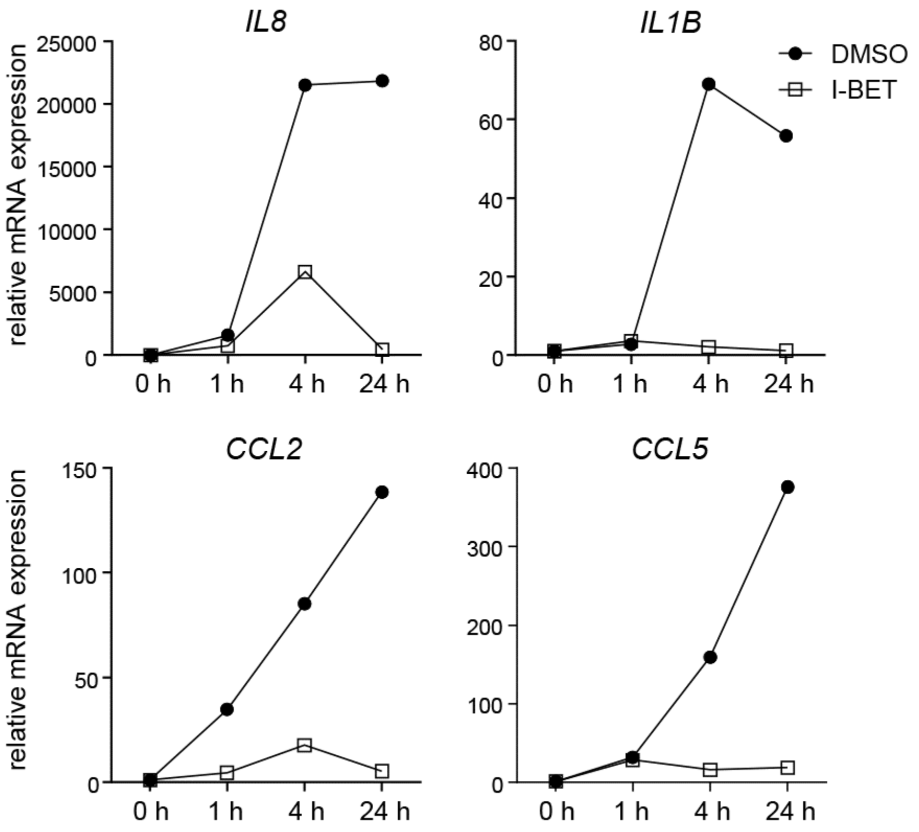

Supplementary Figure 2. I-BET151 suppresses transcriptional induction of inflammatory mediators in gingival fibroblasts (GFs) infected with *P. gingivalis*. qPCR analysis of relative mRNA expression of *IL8*, *IL1B*, *CCL2* and *CCL5*, in GFs treated with DMSO or 1 μM I-BET151 for 30 min prior to infection with *P. gingivalis* (MOI=100) for 1 h, 4 h or 24 h. Data representative of two independent experiments are shown.
